# Topology as a Design Variable for Multiproperty Engineering in Synthesized 4–5–6–8 Carbon Nanoribbons

**DOI:** 10.1021/acsmaterialsau.6c00131

**Published:** 2026-07-30

**Authors:** Djardiel da Silva Gomes, Isaac Macedo Felix, Lucas Lopes Lage, Douglas Soares Galvão, Andrea Latgé, Marcelo Lopes Pereira Junior

**Affiliations:** † Gleb Wataghin Institute of Physics, Department of Applied Physics, 28132 State University of Campinas (UNICAMP), 13083-859 Campinas, São Paulo, Brazil; ‡ Materials Science Postgraduate Program, Faculty UnB Planaltina, 28127 University of Brasília, 73345-010 Brasília, Distrito Federal, Brazil; § Center for Agri-Food Science and Technology, 154624 Federal University of Campina Grande, 58840-000 Pombal, Paraíba, Brazil; ∥ Department of Physics, Federal University of Campina Grande, 58429-900 Campina Grande, Paraíba, Brazil; ⊥ Institute of Physics, 28110 Fluminense Federal University, Av. Litorânea, 24210-340 Niterói, Rio de Janeiro, Brazil; # Center for Computational Engineering and Sciences (CCES), University of Campinas (UNICAMP), 13083-859 Campinas, São Paulo, Brazil; ∇ Department of Electrical Engineering, College of Technology, University of Brasília, 70910-900 Brasília, Distrito Federal, Brazil; ° NanoEngineering Laboratory, College of Technology, University of Brasília, 70910-900 Brasília, Distrito Federal, Brazil

**Keywords:** nonbenzenoid carbon nanoribbons, topology-driven design, quantum transport, electronic properties, mechanical resilience, thermoelectric properties

## Abstract

Nonbenzenoid carbon frameworks expand the design landscape of low-dimensional materials by introducing controlled departures from hexagonal symmetry. Here, we demonstrate that the experimentally realized 4–5–6–8 carbon nanoribbon establishes a topology-driven paradigm for multiproperty engineering rather than representing a simple structural variant of graphene nanoribbons. Combining hybrid density functional theory, parametrized tight-binding, and molecular dynamics within a coherent multiscale framework, we show that the symmetry-broken lattice stabilizes a hierarchy of bonds while remaining in the same energy range with ribbons of comparable width. This geometric organization produces a robust semiconducting state with a hybrid-functional electronic band gap exceeding 1 eV and enables strain to function as a controllable parameter for electronic modulation. Notably, a tight-binding Hamiltonian fitted only at equilibrium, accurately captures the strain-dependent electronic band evolution, indicating that the essential physics is dominated by the topology itself. Mechanical analysis reveals high stiffness with fracture governed by the largest polygonal motifs, demonstrating that geometric asymmetry redistributes stress without compromising structural integrity. In addition, intrinsic phonon scattering suppresses lattice thermal conductance, allowing favorable thermoelectric performance to emerge without extrinsic disorder. The optical response further confirms that nonequivalent ring connectivity reorganizes interband transitions, promoting strong absorption within the visible range and efficient photocarrier generation. These results position the topology as a governing physical parameter capable of coupling elasticity, electronic structure, thermal transport, and optical activity, establishing the 4–5–6–8 nanoribbon as a unified platform for the predictive design of multifunctional carbon materials.

## Graphical abstract

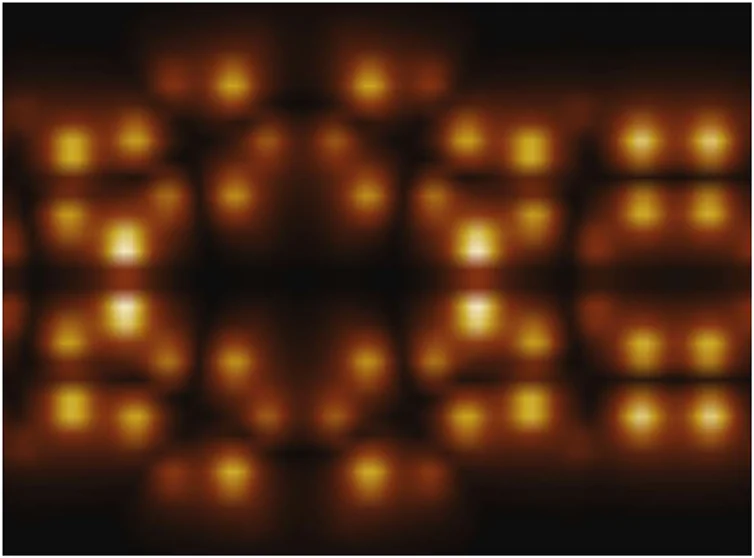

## 1 Introduction

Topology has emerged as a powerful organizing principle to control the physical behavior of low-dimensional materials.^1–3^ In carbon-based systems, where bonding versatility enables a rich diversity of allotropes, altering the polygonal structure of the lattice provides a direct pathway for tailoring electronic states beyond the constraints imposed by hexagonal symmetry.^1,4^ Moving away from the benzenoid paradigm is therefore not merely a structural modification, but a strategy capable of unlocking fundamentally new regimes of quantum behavior.^5,6^

Graphene nanoribbons (GNRs) represent one of the most successful platforms for bandgap engineering through dimensional confinement.^7–9^ Their electronic properties are governed by atomic-scale geometry, including width,^8,10^ edge termination,^11–14^ and boundary conditions,^14–16^ enabling tunable semiconducting behavior suitable for nanoelectronic applications.^17–19^ It should be stressed that, although certain topological analogies can be drawn with graphene nanowiggles (GNWs),^20,21^ such systems constitute a distinct class of nanostructures with different structural origins. Yet, despite these advances, most GNR designs remain rooted in hexagonal networks, where topology acts primarily as a secondary descriptor rather than a deliberate design variable.

A fundamentally different paradigm arises when hexagonal symmetry is intentionally disrupted by the incorporation of nonhexagonal rings.^22–25^ Theoretical studies have long suggested that carbon lattices containing tetragonal, pentagonal, and octagonal motifs can host a wide spectrum of electronic responses, spanning metallic, semimetallic, and semiconducting phases.^24,26–28^ However, the experimental realization of atomically precise architectures embedding multiple polygonal units has historically posed a major challenge.

This limitation was recently overcome with the on-surface synthesis of a carbon nanoribbon composed of 4–5–6–8-membered rings via lateral fusion of polyfluorene chains on Au(111).^29^ High-resolution scanning probe microscopy confirmed its atomic structure and revealed a semiconducting bandgap of approximately 1.4 eV, establishing nonbenzenoid topology as an experimentally accessible design space.^29^ The 4–5–6–8 backbone shares the cyclooctatetraene-like eight-membered ring at the heart of hetero[8]­circulene-based frameworks, whose two-dimensional analogs have attracted considerable attention as ambipolar organic semiconductors.^30,31^ As in those systems, the electronic structure is shaped by the coexistence of aromatic six-membered rings and antiaromatic cyclooctatetraene-like eight-membered rings. First-principles modeling has increasingly been used to anticipate and guide the experimental realization of topologically nontrivial low-dimensional materials.^32^ Despite this breakthrough, a central question remains open, namely whether such topological complexity can serve as a predictive parameter to engineer coupled physical responses rather than merely modifying isolated properties.

Here, we address this question by demonstrating that the deliberate rupture of hexagonal symmetry acts as a topological mechanism for emergent multiphysical behavior in one-dimensional carbon systems. Rather than treating the 4–5–6–8 nanoribbon as an isolated structural realization, we establish the nonbenzenoid topology as a unifying design parameter that simultaneously governs electronic structure, quantum transport, and thermoelectric performance.

To uncover this physics, we have adopted a multiscale computational architecture that bridges atomistic-scale topology with transport-relevant length scales. Hybrid-functional density functional theory (DFT) is employed to obtain an accurate electronic description consistent with experimental observations. These results are subsequently mapped onto a parametrized tight-binding Hamiltonian, enabling large-scale quantum transport calculations while preserving the essential electronic features. The lattice thermal conductivity is further evaluated using reverse nonequilibrium molecular dynamics, allowing charge and heat transport to be assessed within a unified theoretical framework.

Our results reveal that geometric frustration, bond heterogeneity, and symmetry breaking collectively generate strongly coupled electronic and thermal responses, positioning topology not as a passive structural attribute but as an active topological-tune control for functional materials design. By positing nonbenzenoid architectures from synthetic achievements to predictive platforms, this work establishes a conceptual foundation for the rational engineering of next-generation carbon nanostructures.

## 2 Methods

First-principles calculations were carried out within the framework of density functional theory (DFT) using the Spanish Initiative for Electronic Simulations with Thousands of Atoms (SIESTA) code.^33–36^ Structural relaxations and initial electronic analyses employed the Perdew–Burke–Ernzerhof (PBE) generalized gradient approximation,^37,38^ while the hybrid HSE06 functional^39,40^ was adopted to address the well-known electronic band gap underestimation of semilocal functionals. Hybrid-functional calculations were performed using the HONPAS package,^41,42^ which preserves the SIESTA numerical framework.

Core electrons were described by norm-conserving Troullier-Martins pseudopotentials in the Kleinman-Bylander form.^43–45^ A double-ζ polarized basis set was used together with a kinetic energy cutoff of 700 Ry. Brillouin-zone sampling employed a Monkhorst–Pack grid of 20 × 1 × 1.^46^ Atomic positions and lattice vectors were fully relaxed until residual forces were below 0.001 eV/Å and the total energy converged to 10^–5^ eV. Periodic boundary conditions were applied along the ribbon axis, while vacuum regions of 35 Å and 50 Å were introduced along the transverse directions to suppress spurious interactions between periodic images.

Dynamical structural stability was evaluated through phonon dispersion calculations using finite displacements in sufficiently large supercells to eliminate artificial coupling between periodic images.

Thermal structural stability was further assessed via *ab initio* molecular dynamics (AIMD) within the canonical ensemble using a Nosé-Hoover thermostat,^47–49^ with simulations performed at temperatures up to 1500 K.

To access length scales beyond the reach of first-principles methods, large-scale classical molecular dynamics simulations were conducted with the LAMMPS package.^50^ Atomic interactions were modeled using the second-generation reactive empirical bond-order (REBO) potential,^51^ extensively validated for carbon-based nanostructures.^52–56^ The equations of motion were integrated using the velocity-Verlet algorithm,^57^ considering a time step d*t* = 0.1 fs, for a total time of 5.0 ns, and kinetic energy swaps were performed every 2000 time steps. System lengths reached up to ∼200 nm, enabling the accurate description of long-wavelength phonons and realistic mechanical responses.

Lattice thermal transport was computed using the reverse nonequilibrium molecular dynamics (RNEMD) method proposed by Müller-Plathe.^58^ Within this framework, the lattice thermal conductivity σ_ph_ is obtained from Fourier’s law as

1 $$\sigma_{\mathrm{ph}}\left(L_{x}\right)=-\frac{1}{\nabla_{x}T}\left[\frac{\sum_{\mathrm{swaps}}\Delta K}{2A\Delta t}\right]$$

where ∑_swaps_Δ*K* is the total exchanged kinetic energy over the simulation time Δ*t*, ∇_*x*_ *T* is the temperature gradient along the transport direction, and *A* is the effective cross-sectional area of the system. The factor of 2 accounts for the bidirectional heat flux associated with periodic boundary conditions.

The length dependence of the thermal conductivity was analyzed through^59^

2 $$\frac{1}{\sigma_{\mathrm{ph}}\left(L_{x}\right)}=\frac{1}{\sigma_{\mathrm{ph}}^{\infty }}\left(1+\frac{\Lambda }{L_{x}}\right)$$

where σ_ph_^∞^ is the intrinsic thermal conductivity in the diffusive limit and Λ denotes the effective phonon mean free path.

The thermal conductance κ_ph_ (*L*_*x*_) was calculated from the length-dependent thermal conductivity σ_ph_ (*L*_*x*_) using the relation

3 $$\kappa_{\mathrm{ph}}\left(L_{x}\right)=\sigma_{\mathrm{ph}}\left(L_{x}\right)\frac{A}{L_{x}}$$

where *A* is the effective cross-sectional area and *L*_*x*_ is the system length along the transport direction. This definition allows a direct comparison between conductance and conductivity results and is particularly convenient for analyzing size effects in low-dimensional systems. The ballistic lattice thermal conductance κ_ph_^0^ corresponds to the short-length limit of the length-dependent lattice thermal conductance κ_ph_(*L*_*x*_), namely κ_ph_^0^ = lim_*Lx*→0_ κ_ph_(*L*_*x*_).

The optical response was obtained from the complex frequency-dependent dielectric function within the independent-particle approximation

4 $$\varepsilon \left(\omega \right)=\varepsilon_{1}\left(\omega \right)+i\varepsilon_{2}\left(\omega \right)$$

The imaginary part of the dielectric tensor was evaluated within the independent-particle approximation using the momentum-matrix-element formalism appropriate for periodic systems.^60^ It is expressed as

5 $$\varepsilon_{2}^{\alpha \beta }\left(\omega \right)=\frac{4\pi^{2}e^{2}}{\Omega m_{e}^{2}\omega^{2}}\sum_{v,c,k}w_{k}\left\langle \psi_{c,k}\right|{\mathrm{p̂}}_{\alpha }\left|\psi_{v,k}\right\rangle \times \left\langle \psi_{v,k}\right|{\mathrm{p̂}}_{\beta }\left|\psi_{c,k}\right\rangle \delta \left(E_{c,k}-E_{v,k}-\hbar \omega \right)$$

where v and *c* denote valence and conduction states, respectively, *w*_**k**_ is the weight of the **k** point, *p̂*_α_ is the momentum operator along the Cartesian direction α, *m*_e_ is the electron mass, and Ω represents the normalization volume of the simulation cell.

The real part was obtained through the Kramers–Kronig relation^61,62^

6 $$\varepsilon_{1}\left(\omega \right)=1+\frac{2}{\pi }P\int_{0}^{\infty }\frac{\omega^{\prime }\varepsilon_{2}\left(\omega^{\prime }\right)}{\omega^{\prime 2}-\omega^{2}}d\omega^{\prime }$$

ensuring a causal optical response.

Because the nanoribbon is modeled within a three-dimensional periodic supercell with large vacuum regions, the computed dielectric response is artificially reduced by polarization dilution.^63^ To recover the intrinsic quasi-one-dimensional behavior, the dielectric tensor was renormalized according to

7 $$\varepsilon^{1D}\left(\omega \right)=1+\frac{L_{y}L_{z}}{A_{\mathrm{eff}}}\left[\varepsilon^{3D}\left(\omega \right)-1\right]$$

where *L*_*y*_ and *L*_*z*_ are the supercell dimensions perpendicular to the ribbon axis and *A*_eff_ = w*d*_eff_ defines the effective cross-sectional area. Here, *w* is the physical ribbon width, and an effective thickness of *d*_eff_ = 3.35 Å,^64^ corresponding to the graphite interlayer spacing, was adopted. This normalization removes the spurious dependence on vacuum size and enables quantitative comparison with other carbon-based systems.

The complex refractive index *ñ*(ω) = *n*(ω) + *ik*(ω) was obtained from the dielectric function as

8 $$n\left(\omega \right)=\sqrt{\frac{\left|\varepsilon \left(\omega \right)\right|+\varepsilon_{1}\left(\omega \right)}{2}}$$

9 $$k\left(\omega \right)=\sqrt{\frac{\left|\varepsilon \left(\omega \right)\right|-\varepsilon_{1}\left(\omega \right)}{2}}$$

from which the absorption coefficient follows as

10 $$\alpha \left(\omega \right)=\frac{2\omega k\left(\omega \right)}{c}$$

and the reflectivity at normal incidence as

11 $$R\left(\omega \right)={\left|\frac{n\left(\omega \right)-1+\mathrm{ik}\left(\omega \right)}{n\left(\omega \right)+1+\mathrm{ik}\left(\omega \right)}\right|}^{2}$$

The optical conductivity was evaluated through

12 $$\sigma \left(\omega \right)=-i\omega \varepsilon_{0}\left[\varepsilon \left(\omega \right)-1\right]$$

while the energy-loss function

13 $$L\left(\omega \right)=I\left[-\frac{1}{\varepsilon \left(\omega \right)}\right]=\frac{\varepsilon_{2}\left(\omega \right)}{\varepsilon_{1}^{2}\left(\omega \right)+\varepsilon_{2}^{2}\left(\omega \right)}$$

was used to identify collective electronic excitations.

All spectra were computed considering light polarized along the longitudinal direction of the ribbon axis, thus capturing the intrinsic optical anisotropy of the quasi-one-dimensional lattice. A Gaussian broadening of 0.15 eV was introduced to mimic finite excited-state lifetimes and to smooth discrete interband transitions near the band edges.

The mixed-polygon connectivity of the 4–5–6–8 nanoribbon supports a quasi-one-dimensional electronic landscape in which single transport channels dominate the low-energy response. This regime is conveniently described by a single-orbital tight-binding Hamiltonian

14 $$H=\sum_{i}\epsilon c_{i}^{†}c_{i}+\sum_{\left\langle i,j\right\rangle }t_{\mathrm{ij}}\left(c_{i}^{†}c_{j}+H.c.\right)$$

where ϵ is the (site-uniform) on-site energy, *c*_*i*_^†^ (*c*_*i*_) is the creation (annihilation) operator at site *i*, and *t*_*ij*_ is the hopping integral between sites *i* and *j*, restricted to pairs ⟨*i*, *j*⟩ within the cutoff defined below. The hopping decays exponentially with bond length as

15 $$t_{\mathrm{ij}}=t_{1}e^{-\beta \left(r_{\mathrm{ij}}/d_{\min}-1\right)}$$

where *t*_1_ is the hopping at the first-nearest-neighbor distance, *r*_*ij*_ is the bond length between sites *i* and *j*, *d*_min_ = 1.39 Å is the nearest-neighbor reference distance, and β is a fitting parameter controlling the spatial range of the hopping. Since *r*_*ij*_/*d*_min_ ≥ 1 beyond the first coordination shell, smaller values of β extend the number of neighbors with non-negligible hopping. The parameters were adjusted against the DFT band dispersion, yielding ϵ = −0.9 eV, *t*_1_ = −3.85 eV, and β = 2.8. The same parametrization scheme has been applied to nonbenzenoid carbon allotropes, including biphenylene-based systems^65,66^ and organic chains.^67^

Quantum transport properties are investigated using the Green’s function G(E) within the Landauer formalism^68^ defined as

16 $$G\left(E\right)={\left[EI-H-\Sigma_{L}\left(E\right)-\Sigma_{R}\left(E\right)\right]}^{-1}$$

where Σ_*L*_(*E*) and Σ_*R*_(*E*) are the self-energies of the left and right electrodes, respectively, evaluated at energy *E*. The total density of states (DOS) is obtained from DOS(E)=−(1/π)Im{Tr[G(E)]} . The transmission function T(E) is given by^68^

17 $$T\left(E\right)=\mathrm{Tr}\left[\Gamma_{L}\left(E\right)G\left(E\right)\Gamma_{R}\left(E\right)G^{†}\left(E\right)\right]$$

where Γ_*L*_(*E*) and Γ_*R*_(*E*) are the broadening matrices of the left and right electrodes. In this work, the leads are taken to be structurally identical to the scattering region, as illustrated in Figure 1(a).

**Figure 1:**
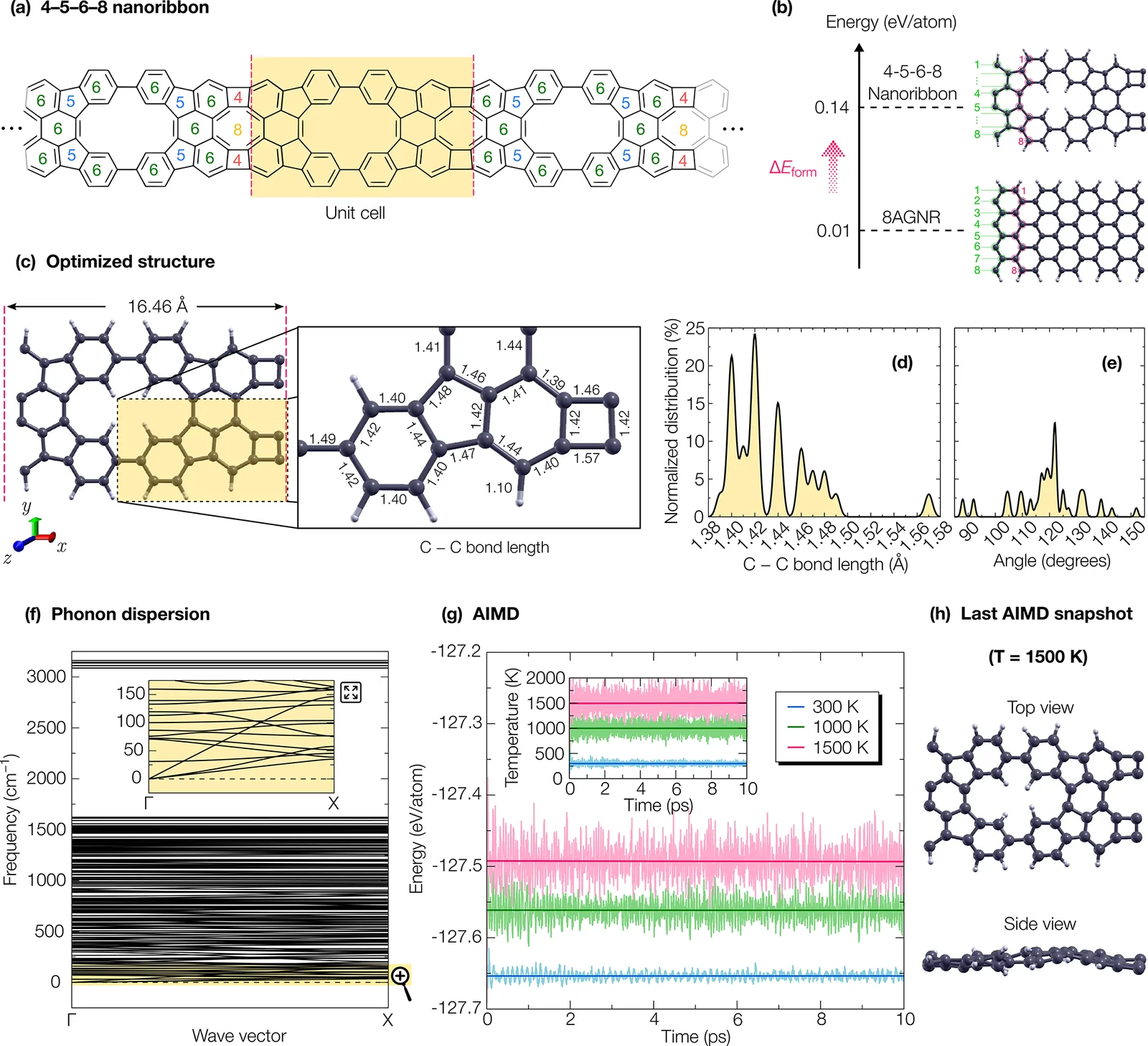
Structure and stability of the 4–5–6–8 carbon nanoribbon. (a) Atomic structure of the ribbon backbone. (b) Formation energy per atom referenced to graphene and molecular hydrogen for the 4–5–6–8 nanoribbon and the width-matched 8AGNR, with the eight carbon atoms spanning the transverse width labeled on each structure. (c) Optimized geometry with representative C–C bond lengths. (d, e) Statistical distributions of bond lengths and bond angles. (f) Phonon dispersion. (g) Total energy from *ab initio* molecular dynamics at multiple temperatures. (h) Final AIMD snapshot at 1500 K.

The electronic current across the scattering region reads

18 $$I=\frac{2e}{h}\int dET\left(E\right)\times \left[f_{L}\left(E,\mu ,T\right)-f_{R}\left(E,\mu ,T\right)\right]$$

with *f*_*L*,*R*_(*E*, μ, *T*) = [1 + e^(*E*–μ_*L*,*R*_)/*k*_*B*_ *T*^]^−1^ being the Fermi–Dirac distributions of left and right leads, respectively. An applied bias, V, shifts the left and right chemical potentials as μ_*L*,*R*_ = *E*_F_ ± e*V*/2, with *E*_F_ being the electrode Fermi energy. To pursue the spatial distribution of electronic transmission, we employ a local transmission coefficient *T*_*ij*_^α^(*E*) which computes the transmission between *j* and *i* sites, and leads α = *L*, *R* for a given energy,^69^ namely

19 Tijα(E)=2Im{[G(E)ΓαG†(E)]jiHij}

Once we compute the electronic transmission T(E) for *T* = 0 K, the electronic conductance *G*(μ, *T*), Seebeck coefficient *S*(μ, *T*), power factor PF­(μ, *T*), and electronic thermal conductance κ_e_(μ, *T*) can be obtained in terms of standard generalized transport coefficient $L_{n}\left(\mu ,T\right)$ , defined as^70,71^

20 $$L_{n}\left(\mu ,T\right)=-\frac{2}{h}\int dET\left(E\right)\times {\left(E-\mu \right)}^{n}\partial_{E}f\left(E,\mu ,T\right)$$

The conductance *G*(μ, *T*), Seebeck coefficient *S*(μ, *T*), power factor PF­(μ, *T*), and electronic thermal conductance κ_e_(μ, *T*) are then obtained as

21 $$G\left(\mu ,T\right)=e^{2}L_{0}\left(\mu ,T\right)$$

22 $$S\left(\mu ,T\right)=-\frac{1}{eT}\frac{L_{1}\left(\mu ,T\right)}{L_{0}\left(\mu ,T\right)}$$

23 $$\mathrm{PF}\left(\mu ,T\right)=S{\left(\mu ,T\right)}^{2}G\left(\mu ,T\right)$$

24 $$\kappa_{e}\left(\mu ,T\right)=\frac{1}{T}\left[L_{2}\left(\mu ,T\right)-\frac{L_{1}{\left(\mu ,T\right)}^{2}}{L_{0}\left(\mu ,T\right)}\right]$$

Finally, the thermoelectric figure of merit is given by

25 $$\mathrm{ZT}=\frac{G\left(\mu ,T\right)S{\left(\mu ,T\right)}^{2}T}{\kappa_{e}\left(\mu ,T\right)+\kappa_{\mathrm{ph}}\left(T\right)}$$

where κ_ph_(*T*) is the phononic contribution to the thermal conductance, treated as a temperature-dependent quantity decoupled from the electronic chemical potential.

These quantities provide the framework for analyzing the thermal, electronic, and structural stability of the 4–5–6–8 nanoribbon.

## 3 Results and Discussion

We begin by examining the structural consequences imposed by the nonbenzenoid topology, since the lattice’s geometrical topology ultimately constrains the stability window within which electronic and transport phenomena emerge. Figure 1 provides a multiscale structural description of the 4–5–6–8 nanoribbons, connecting atomic tiling, bonding statistics, lattice dynamics, and finite-temperature behavior. Rather than representing a perturbed/distorted graphene ribbon, this system defines a topologically distinct carbon phase in which tetragons and octagons are periodically embedded into a predominantly *sp* ^2^ network.

The backbone shown in Figure 1(a) reproduces the structural motif resolved experimentally through on-surface synthesis, where scanning probe measurements confirmed the periodic arrangement of 4-, 5-, 6-, and 8-membered rings. This synthetic route demonstrates that the ribbon is not a metastable theoretical construct but an experimentally accessible topology stabilized through controlled cyclodehydrogenation reactions. Establishing whether such a symmetry-broken lattice remains energetically competitive with graphene-derived nanoribbons is therefore an important and necessary analysis.

To quantify the relative structural stability, we evaluated the cohesive energy per atom, defined as

26 $$E_{\mathrm{cohe}}=\frac{E_{\mathrm{tot}}-\sum_{i}n_{i}E_{i}^{\mathrm{atom}}}{N}$$

where *E*_tot_ is the total energy of the relaxed unit cell, *E*_*i*_^atom^ is the energy of an isolated atom of species *i* evaluated in its spin-polarized ground state, *n*_*i*_ is its multiplicity, and *N* is the total number of atoms. Normalized per carbon atom, which removes the effect of the different hydrogen fractions, *E*_cohe_ of the 4–5–6–8 ribbon (−8.76 eV per carbon atom) is nearly degenerate with that of the width-matched 8AGNR (−8.80 eV per carbon atom), a difference below 0.05 eV, so the carbon network is bound as strongly as that of the hexagonal counterpart. Per projected area, *E*_cohe_ is about 15% lower for the ribbon (−2.64 eV Å^–2^) than for the 8AGNR (−3.08 eV Å^–2^), a reduction that follows from the lower areal atomic density of the porous lattice, 0.394 against 0.44 Å^–2^, rather than from any weakening of the individual bonds.

To measure the energetic cost of the nonhexagonal topology relative to a common reference, we further evaluated the formation energy

27 $$E_{\mathrm{form}}=\frac{E_{\mathrm{tot}}-n_{C}\mu_{C}-n_{H}\mu_{H}}{N}$$

where *n*_C_ and *n*_H_ are the numbers of carbon and hydrogen atoms, and μ_C_ = *E*_graphene_/2 and μ_H_ = *E*_H_2__/2 are the chemical potentials of a carbon atom in graphene and of a hydrogen atom in molecular hydrogen. Figure 1(b) compares the formation energy of the 4–5–6–8 ribbon and the width-matched 8AGNR, both computed within the same methodology. It is +0.14 eV per atom (+0.18 eV per carbon atom) for the ribbon, whereas the 8AGNR lies essentially at the graphene reference, +0.01 eV per atom, as expected for a graphene-derived armchair ribbon terminated by hydrogen. This modest topological penalty is comparable to that of phagraphene, about 0.2 eV per atom,^26^ and below that of the biphenylene network, about 0.5 eV per atom,^23^ supporting the energetic competitiveness of the lattice.

This interpretation aligns well with first-principles simulation results reported by Mortazavi,^27^ who identified bond heterogeneity as an intrinsic structural feature of pristine 4–5–6–8 nanoribbons. In that work, nitrogen substitution at the ribbon edges produced a measurable contraction of the lattice and redistributed the mechanically critical C–C bonds surrounding the nonbenzenoid junctions, demonstrating that the edge’s chemistry directly couples to the global strain landscape. The agreement between these trends and current structural metrics supports the conclusion that the observed bonding hierarchy arises from topology rather than computational artifacts.

The optimized structure shown in Figure 1(c) clarifies how the lattice changes balance the competing angular constraints imposed by mixed polygons. The unit-cell length of approximately 16.46 Å defines the longitudinal periodicity, while the highlighted region reveals a broad distribution of C–C bond lengths ranging from about 1.38 to 1.58 Å. This spread of Δ_CC_ ≈ 0.20 Å exceeds the essentially uniform bonding of graphene by more than an order of magnitude, and the bond dispersion of narrow armchair ribbons, which is largely edge-driven and of the order of 0.07 Å,^72^ by roughly 3-fold. It is the structural fingerprint that propagates into the asymmetric spectral weight of the density of states and into the intrinsic phonon scattering discussed below. In contrast to graphene and narrow AGNRs, which typically exhibit nearly uniform bonding, the present topology redistributes strain across multiple ring types, generating localized regions of compression and extension that collectively stabilize the network. This bonding hierarchy has a chemical origin in the competition between the aromatic stabilization of the hexagonal rings and the local antiaromatic character of the cyclooctatetraene-like octagon. The aromatic rings favor shorter and more uniform bonds, whereas the antiaromatic octagon accommodates the longer and weaker bonds that terminate the distribution, and the same competition conditions the electronic gap and the strain response discussed below.

The statistical distributions in Figure 1(d),1(e) provide a fingerprint of these structural accommodations. The bond-length histogram exhibits multiple maxima rather than the single dominant peak characteristic of hexagonal carbon, confirming that structural heterogeneity is topologically enforced. The angular distribution is equally revealing. The absence of a pronounced peak near 90° indicates that the four-membered rings do not adopt ideal rectangular geometries but instead undergo angular distortion that partially relieves strain through coupling with adjacent pentagon and octagon rings. Similar distortions and the identification of specific load-bearing bonds have been reported before in the literature,^27^ further supporting the view that the mechanical resilience in these ribbons arises from cooperative bond rearrangements around the nonbenzenoid cores.

The comparison with the 8AGNR highlights the structural implications of symmetry breaking. Whereas the honeycomb lattice tightly constrains geometric degrees of freedom in armchair ribbons, the 4–5–6–8 topology introduces an internal strain landscape while preserving the dominant *sp* ^2^ character, as evidenced by the persistence of bond angle distributions around 120°. The resulting backbone is therefore structurally heterogeneous yet still electronically *sp* ^2^-type conjugated, a combination difficult to achieve through conventional graphene patterning.

The dynamical structural stability is confirmed by the phonon dispersion presented in Figure 1(f), where no imaginary frequencies are present throughout the entire Brillouin zone, indicating that the ribbon resides at a true minimum of the potential-energy surface. The highest-frequency optical modes extend beyond approximately 3000 cm^–1^ and are primarily associated with C–H stretching vibrations localized at the ribbon edges, while intermediate-frequency modes originate from in-plane C–C stretching within the backbone. The absence of soft modes indicates that the geometric frustration introduced by the nonbenzenoid topology does not lead to vibrational instability.

Thermal robustness was further assessed through ab initio molecular dynamics.^73^ As shown in Figure 1(g), the total energy remains stable (within the limit of the thermal fluctuations) at temperatures up to 1500 K. They fluctuate around stable average values without signatures of bond break or large-scale structural reconstructions. The final configuration displayed in Figure 1(h) confirms the preservation of the backbone, with only slightly out-of-plane structural corrugations, which are typical of atomically thin materials. Comparable thermal stability of both pristine and chemically modified variants has been reported,^27^ supporting the interpretation that the mixed-ring topology defines a mechanically robust carbon phase once formed.

From an experimental standpoint, this stability is particularly relevant because the synthesis protocol involves annealing steps that activate polymer fusion and cyclodehydrogenation. The ability of the ribbon to withstand substantially higher simulated temperatures suggests that the nonbenzenoid backbone constitutes a resilient metastable configuration compatible with experimentally accessible growth conditions.

Taken together, Figure 1 demonstrates that the 4–5–6–8 topology simultaneously accommodates geometric distortion, energy viability, and dynamical structural stability. Breaking hexagonal symmetry does not compromise the lattice integrity. Instead, it generates a hierarchy of bonds and angles that stabilizes the structure while introducing internal degrees of freedom absent in conventional graphene nanoribbons.

These experimentally realizable, thermally robust structural features provide the physical foundation for understanding the emergent electronic and transport properties discussed next. In conjugated carbon systems, the electronic structure is inseparable from the underlying bonding network. Consequently, the strain redistribution and bond hierarchy imposed by the mixed-ring topology are expected to reshape the π-electron manifold directly. Figure 2 examines this connection by combining hybrid-functional electronic structure, tight-binding reconstruction, and real-space transport analysis within a unified framework. Rather than behaving as a perturbed/distorted armchair graphene nanoribbon, the 4–5–6–8 lattice defines an electronically distinct regime in which symmetry breaking becomes an active parameter for electronic band engineering.

**Figure 2:**
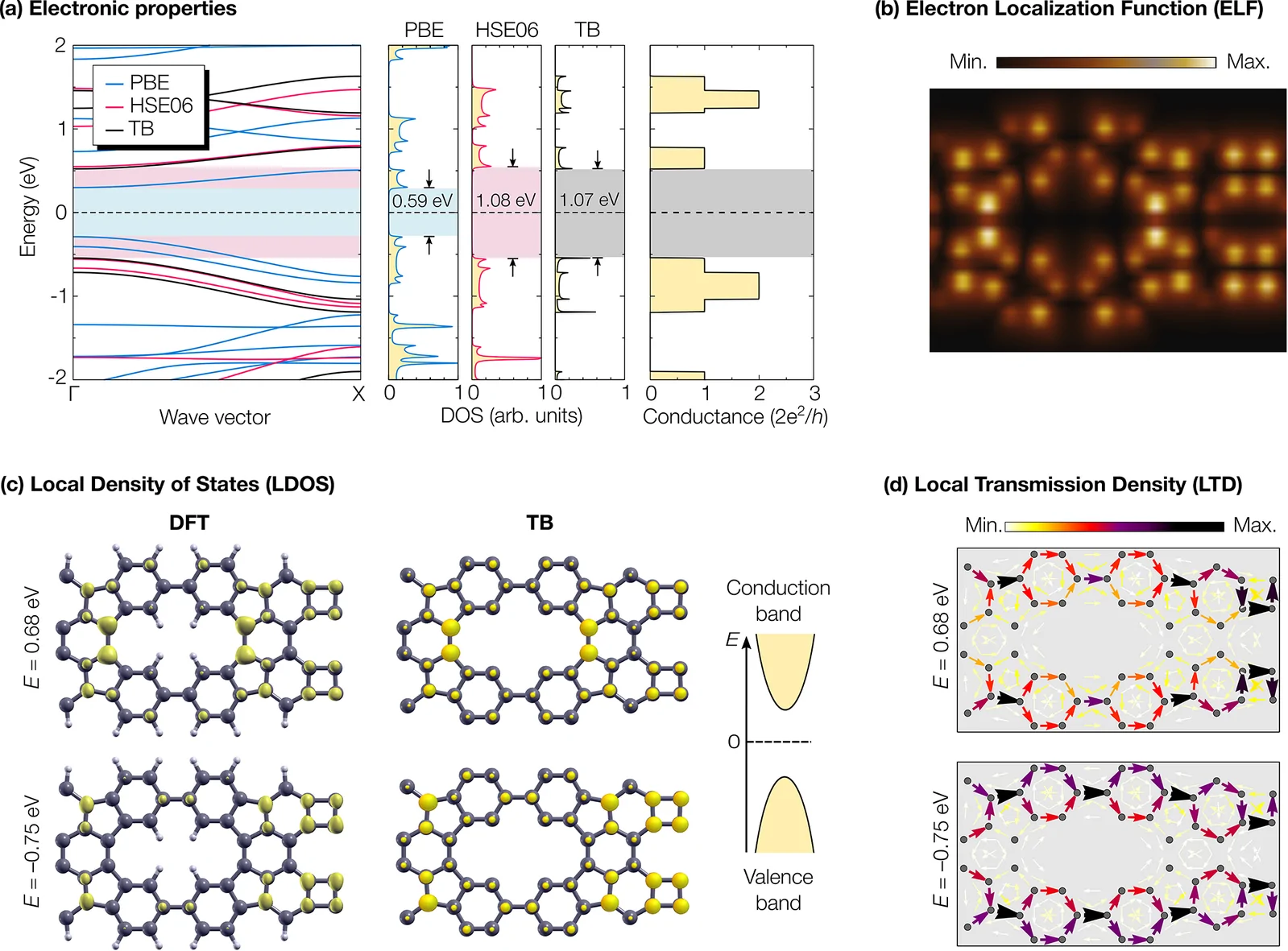
Electronic and transport properties of the 4–5–6–8 nanoribbon. (a) Band structure (left), density of states (middle), and quantum conductance (right) compared between PBE (blue), HSE06 (red), and tight-binding (TB, black) methods. (b) Electron localization function map. (c) Local density of states from DFT and TB. (d) Local transmission density. Panels (c) and (d) are evaluated at the valence-band peak (*E* = −0.75 eV) and conduction-band valley (*E* = 0.68 eV).

All methods reveal that the electronic band structures shown in Figure 2(a) are of semiconductors, while simultaneously exposing the importance of exchange-correlation treatment. The PBE functional predicts an electronic band gap of approximately 0.59 eV, whereas the screened hybrid functional increases this value to about 1.08 eV, correcting the typical gap underestimation of semilocal approximations and placing the electronic scale closer to experimentally relevant energies. Hybrid-functional calculations previously reported for this topology exhibit the same qualitative trend,^27,31^ reinforcing that the gap opening is not a methodological artifact but a robust electronic feature of the nonbenzenoid backbone. The tight-binding Hamiltonian, parametrized directly from the HSE06 dispersion, reproduces the band gap value of the hybrid functional (1.07 eV) while preserving the curvature of the frontier bands. This hierarchical consistency demonstrates that the multiscale strategy is not a redundant stacking of techniques, but a physically necessary pathway that transfers hybrid-functional accuracy to transport-accessible length scales.

From the perspective of graphene nanoribbon physics, the magnitude and origin of this gap are particularly instructive. At the matched width, our PBE and HSE06 calculations give the conventional 8AGNR only a small gap, 0.21 and 0.29 eV, consistent with the near-metallic 3p + 2 family and with the edge-bond relaxation that opens it in an otherwise metallic family.^7^ The 4–5–6–8 ribbon, by contrast, sustains a robust gap of 0.59 eV within PBE and 1.08 eV within HSE06, a 3-fold to 4-fold widening at fixed width. The 4–5–6–8 ribbon cannot be mapped onto any of the canonical armchair families (3p, 3p + 1, 3p + 2), because the periodic insertion of nonhexagonal rings breaks the translational symmetry that underpins that classification. The different geometry leads to a wider band gap, since orbital overlap reorganization is driven by angular frustration. Thus, the lattice topology, rather than lateral confinement, governs the gap and reduces the transport channels relative to the width-matched 8AGNR near the Fermi level. These quantities are intrinsic to the specific transverse width of the ribbon, since in quasi-one-dimensional carbon systems the lateral confinement and the finite fraction of edge atoms jointly control the magnitude of the gap and of the elastic and thermal responses, which requires a meaningful comparison with an armchair of matched width.

The density of states in Figure 2(a) further highlights this departure from conventional armchair behavior. Instead of the symmetric van Hove singularities characteristic of graphene nanoribbons, the spectrum exhibits uneven spectral weight near the band edges, indicating that the valence and conduction states originate from nonequivalent bonding environments. This asymmetry is a direct electronic manifestation of the bond heterogeneity established in Figure 1, confirming that structural frustration propagates into the low-energy electronic landscape. To confirm that this nonmagnetic semiconducting solution is the ground state, spin-polarized calculations initialized from ferromagnetic, antiferromagnetic, and edge- and ring-centered broken-symmetry configurations were performed. All converge to the nonmagnetic state, with vanishing net and local moments and energies indistinguishable from the nonmagnetic value, so the semiconducting nonmagnetic ground state is robust despite the geometric frustration of the tetragonal and octagonal rings.

Insight into the orbital organization is provided by the electron localization function shown in Figure 2(b). Regions of strong localization trace the shorter C–C bonds surrounding pentagon-hexagon junctions, whereas comparatively depleted charge density appears near the more weakly bonded segments associated with the larger rings. The resulting pattern indicates that π electrons remain globally conjugated but are spatially modulated by the internal strain field. Such redistribution establishes preferential pathways for carrier motion while preserving electronic continuity along the backbone.

Hybrid-functional studies of chemically modified variants of this ribbon have previously shown that nitrogen substitution alters both the electronic band gap and charge distribution by introducing electron-rich centers that locally reshape the frontier states without significantly disrupting conjugation.^27^ The fact that comparable electronic anisotropy already emerges in the pristine structure indicates that topology alone places the system near a regime of electronically tunable frustration, with heteroatom incorporation acting as a secondary refinement rather than a prerequisite for band engineering.

Real-space projections of the frontier states, presented in Figure 2(c), confirm that both band edges are dominated by *p*_*z*_ orbitals forming an extended π network across the ribbon interior. Enhanced amplitudes are consistently observed near the nonbenzenoid junctions, identifying these regions as electronic hot spots where curvature in the bonding topology spatially concentrates electronic wave functions. This behavior establishes a meaningful parallel with experimental scanning-probe observations, which revealed that states near the band edges propagate along the ribbon backbone instead of being localized exclusively at the edges, consistent with a bulk-like conduction channel embedded within a quasi-one-dimensional geometry.

The transport consequences of this orbital structure are captured by the conductance profile in Figure 2(a). Quantized steps emerge as discrete propagating modes become available, and the rapid increase in conductance indicates that multiple channels are opened within a narrow energy window. In contrast to a 8AGNR, whose transmission is largely dictated by width-controlled subbands, the present ribbon benefits from internally generated conduction pathways arising from topological heterogeneity. The mixed-ring lattice, therefore, behaves as a self-patterned electronic medium in which geometry preconfigures transport.

This interpretation is reinforced by the local density of states and transmission maps. The LDOS distributions in Figure 2(c) reveal that frontier states remain spatially continuous, avoiding the strong edge confinement that can limit mobility in narrow graphene nanoribbons. Meanwhile, the local transmission density shown in Figure 2(d) traces percolating current pathways that adapt to the polygonal arrangement, bending around pentagon and octagon rings while maintaining global directionality. Carrier flow is therefore redirected rather than suppressed by the broken symmetry.

The electronic response thus emerges directly from the symmetry-broken bonding network introduced by the 4–5–6–8 topology. By redistributing orbital overlap across nonequivalent polygons, the lattice reshapes the density of states and promotes spatially extended transport channels that are inaccessible within purely hexagonal ribbons. In this framework, topology ceases to be a geometric descriptor and instead becomes a governing physical parameter that couples band formation to carrier propagation, establishing a robust platform for multiproperty engineering in low-dimensional carbon systems.

Figure 3(a) shows that the 4–5–6–8 nanoribbon exhibits a well-defined linear elastic regime, from which a Young’s modulus of 419.4 ± 2.4 GPa is estimated. Although smaller than the stiffness typically reported for pristine graphene, this magnitude firmly places the ribbon within the class of mechanically resilient carbon nanostructures. The reduced modulus should be inferred as a direct consequence of bond heterogeneity rather than structural fragility. While hexagonal lattices distribute stress nearly uniformly, the mixed-ring topology introduces bonds with distinct stiffnesses, enabling the structure to redistribute elastic energy through multiple deformation pathways, but less effectively than the hexagonal topology.

**Figure 3:**
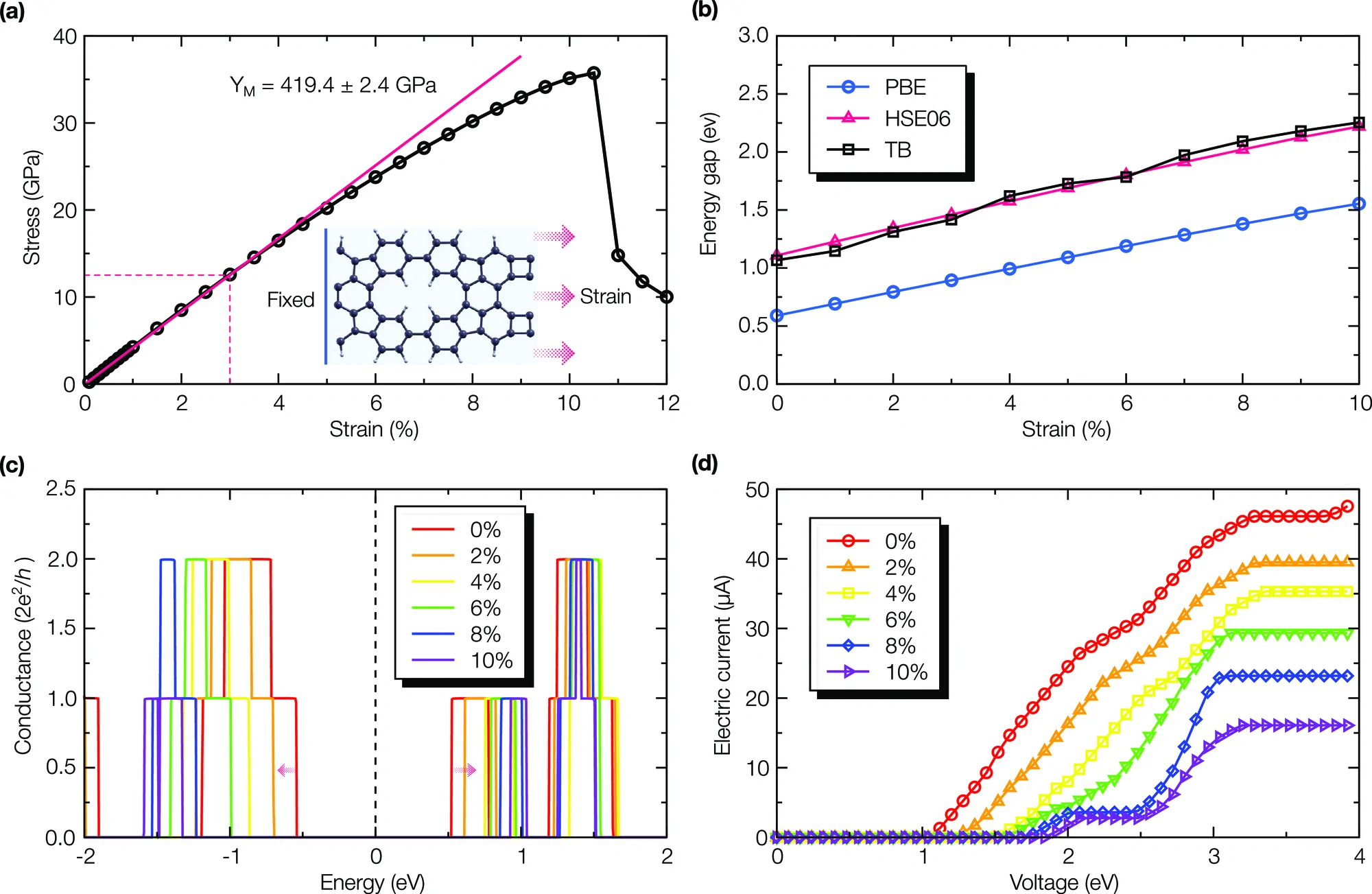
Mechanical and electronic response of the 4–5–6–8 nanoribbon under uniaxial strain at *T* = 0 K. (a) Stress–strain curve from DFT. (b) Energy gap versus strain, comparing PBE (blue), HSE06 (red), and TB (black). (c) Electronic conductance and (d) electric current as a function of bias voltage, computed with TB for strain levels from 0% to 10%.

The fracture mechanism provides a particularly clear demonstration of how topology determines the mechanical response. Structural failure nucleates at the largest interior ring, precisely where the density of load-bearing bonds is lowest and angular distortion is highest. First-principles simulations have shown that two symmetric C–C bonds located at the center of this nanopore dominate the tensile strength, effectively defining the mechanical limit of the ribbon fracture. Nitrogen substitution^27^ near these bonds reduces the tensile strength from approximately 40 GPa to approximately 33 GPa, confirming that local electronic perturbations directly weaken the primary stress-transfer channel while leaving the global framework intact. Thermal simulations further indicate that both pristine and nitrogen-terminated ribbons remain structurally stable at elevated temperatures, demonstrating that the predicted failure mode reflects intrinsic topology rather than thermally activated degradation.

This behavior contrasts with the fracture patterns typically observed in armchair graphene nanoribbons, where rupture frequently begins at edges or defect sites. In the current system, failure is encoded in the internal geometry itself. Symmetry breaking, therefore, does not introduce mechanical weakness. Instead, it spatially programs the locus of rupture, transforming topology into a predictive descriptor of mechanical robustness.

Once mechanical integrity is established, strain can be viewed not as a destructive perturbation but as a controllable thermodynamic variable that can reshape the electronic structure. Figure 3(b) reveals a monotonic widening of the band gap with increasing tensile strain for all methods. The hybrid functional shifts the electronic scale upward relative to the PBE, while the tight-binding Hamiltonian, parametrized only once from the unstrained hybrid dispersion, reproduces the strain dependence with remarkable accuracy. This agreement indicates that the governing physics is encoded in the connectivity of the nonhexagonal backbone rather than in functional-specific details, allowing hybrid-level fidelity to propagate into transport-relevant regimes without the need of iterative refitting.

In standard graphene nanoribbons, the electronic band gap modulation is largely interpreted through quantum confinement, with the band gap scaling inversely with ribbon width. The 4–5–6–8 lattice exhibits a distinct regime. Because the frontier states are already influenced by angular frustration and bond hierarchy, strain acts on a prestructured orbital network. Topology and mechanical deformation, therefore, act cooperatively, establishing a dual control mechanism for electronic band engineering, not available in purely hexagonal systems.

The transport response confirms this interpretation. Quantized conductance plateaus in Figure 3(c), given by 1 and 2 units of quantum conductance, shift systematically with strain, tracking the displacement of the band edges, while the current–voltage characteristics in Figure 3(d) exhibit progressively lower currents as the electronic band gap widens. Tensile loading thus provides a continuous means of regulating carrier injection without disrupting coherent transport channels. The resulting changes are reflected in the electric current, which requires an additional 1 eV at 10% strain to activate the response compared to the unstrained case. The decreasing current values as the strain increases from 0% to 10%, resulting from a reduction in available transport states, are clearly shown in the current values. The saturation current at higher voltages drops from 45 to 15 μA.

Mechanical deformation, electronic structure, and carrier transport consequently emerge as interdependent manifestations of the same symmetry-broken bonding architecture. The 4–5–6–8 topology does not merely withstand strain, it converts it into a predictable electronic response while preserving structural resilience. Topology, therefore, advances beyond a geometric motif to serve as a functional design parameter that couples elasticity to electronic band engineering in low-dimensional carbon materials.

If topology simultaneously governs electronic dispersion and mechanical adaptability, the same structural principle should also regulate energy flow. Thermoelectric performance provides a stringent test of this hypothesis because it depends on the delicate balance between carrier mobility and phonon-mediated heat transport. Figure 4 addresses this interplay by combining lattice thermal conductance obtained from molecular dynamics with tight-binding electronic transport, thereby extending the multiscale framework from atomistic stability to device-relevant thermodynamic regimes.

**Figure 4:**
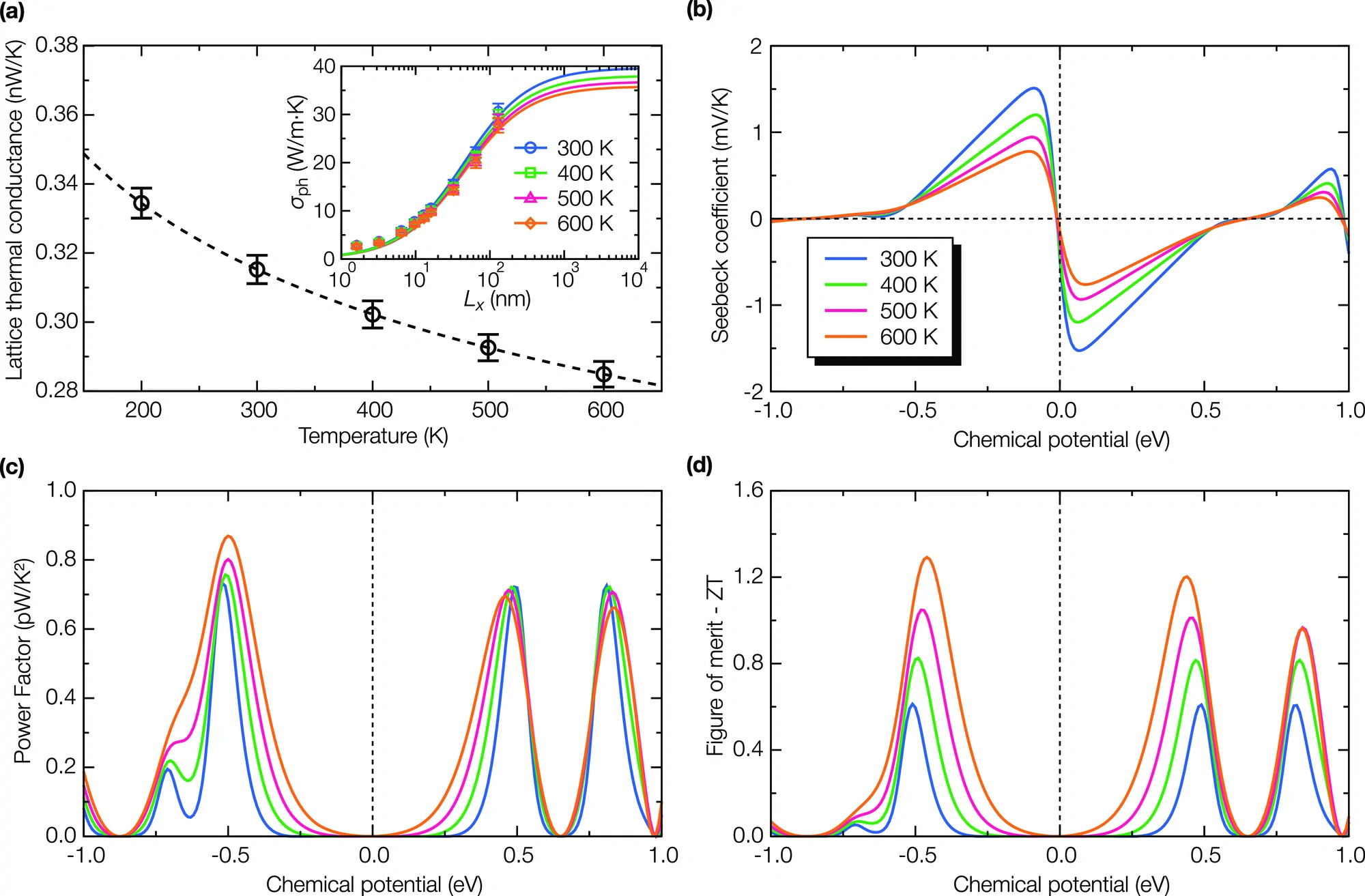
Thermal and thermoelectric transport properties. (a) Lattice thermal conductance (κ_ph_) as a function of temperature, calculated using molecular dynamics. The inset shows the phonon thermal conductivity (σ_ph_) as a function of length (*L*). (b) Seebeck coefficient, (c) Power factor, and (d) thermoelectric figure of merit (ZT) plotted as a function of chemical potential. The curves represent different temperatures ranging from 300 to 600 K.

Figure 4(a) reveals a monotonic reduction of the ballistic lattice thermal conductance (κ_ph_^0^) with increasing temperature, a parameter derived from the length-dependent thermal conductivity scaling through eq 3. This behavior underscores that the topological framework, which was previously shown to govern lattice stability and the electronic response to strain, serves as the fundamental regulator of heat transport. The observed temperature-dependent trend reflects the influence of bond heterogeneity on lattice dynamics, defining the upper bound for energy transport prior to the onset of diffusive scattering.

The inset of Figure 4(a) depicts the thermal conductivity of the 4–5–6–8 nanoribbon as a function of the characteristic ribbon length, σ_ph_(*L*_*x*_), across various temperatures. By applying an extrapolation scheme for the infinite-length limit based on the ballistic-to-diffusive transition (eq 2), the intrinsic thermal conductivity σ_ph_^∞^ was found to be 39.7 ± 1.6 W/mK at 300 K. This decrease is comparable to that observed in GNRs with pores,^9^ isotopic doping,^74^ chemisorption functionalization,^75^ and Stone-Thrower-Wales defects.^76^ To provide a systematic quantitative benchmark and isolate the effect of hexagonal symmetry breaking, identical simulations were performed for a width-matched 8AGNR, which exhibited a significantly higher intrinsic conductivity of 207.0 ± 23.2 W/mK at 300 K, in agreement with previous reports.^75,77,78^

A direct comparison confirms that the periodic topological defects in the 4–5–6–8 nanoribbon suppress thermal conductivity by more than 80% relative to the pristine 8AGNR. This pronounced decrease is attributed to enhanced phonon scattering at the pentagonal and octagonal rings, which act as intrinsic scattering centers.

Figure 4(a) reveals a monotonic reduction of the lattice thermal conductance with increasing temperature, reflecting the progressive activation of phonon–phonon scattering channels. More revealing than the temperature trend itself is the magnitude of the thermal response. In pristine graphene, thermal conductivity can exceed several thousand W m^–1^ K^–1^, a consequence of long phonon mean free paths and the absence of strong scattering centers.^79^ Such efficient heat transport, while beneficial for thermal management, is fundamentally detrimental to thermoelectric efficiency because it suppresses the figure of merit.^80^

The mixed-ring backbone alters this paradigm at the structural level. By redistributing bond lengths and introducing angular frustration across nonequivalent polygons, the 4–5–6–8 topology generates intrinsic phonon scattering without relying on extrinsic disorder, edge roughness, or isotope engineering. Heat transport is therefore attenuated as a direct consequence of lattice geometry. Rather than treating phonon suppression as a postsynthetic optimization strategy, the present architecture embeds it into the bonding network itself.

The inset of Figure 4(a) further clarifies the transport regime. The length dependence of σ_ph_ captures the expected crossover from ballistic to diffusive propagation, enabling extrapolation toward the intrinsic conductivity limit. This behavior closely parallels that of graphene nanoribbons, in which phonon mean free paths strongly influence size-dependent thermal transport, yet the absolute scale is reduced here by the lattice’s structural complexity. The topology, therefore, operates as an internal regulator of heat flow, effectively shortening the phonon propagation length while preserving crystalline order.

Suppressing lattice thermal conductance alone does not guarantee favorable thermoelectric behavior. The decisive factor is whether electronic transport remains sufficiently robust. The Seebeck response shown in Figure 4(b) provides strong evidence that this condition is satisfied. The pronounced antisymmetric profile around the chemical potential reflects the semiconducting character established earlier, while the increase in peak magnitude at lower temperatures follows the sharpening of the electronic distribution near the band edges.

The power factor displayed in Figure 4(c) consolidates this picture. Its maxima occur near the band edges, where rapid variation of the transmission function boosts thermopower while maintaining appreciable electrical conductance. Importantly, the temperature evolution indicates that carrier transport remains stable across the thermal explored window, reinforcing that the electronic backbone is not compromised by the structural distortions that suppress phonons.

These combined effects culminate in the figure of merit shown in Figure 4(d). The emergence of elevated ZT values should not be interpreted as an isolated transport outcome, but as the macroscopic expression of a deeper organizing principle. Once hexagonal symmetry is relaxed, the lattice acquires internal degrees of freedom that mediate charge and heat transport along partially decoupled channels. Electronic states remain sufficiently delocalized to sustain transport, whereas phonon propagation is intrinsically disrupted by bond hierarchy and polygonal diversity. In fact, our findings contrast with the behavior of conventional two-dimensional graphene, whose gapless nature results in minimal thermopower.

The 4–5–6–8 nanoribbon reaches a room-temperature figure of merit of about 0.6, well above the values reported for pristine armchair graphene nanoribbons, which are typically limited to ZT ≈ 0.05 to 0.2 by their high lattice thermal conductance.^81^ This enhancement follows from a higher Seebeck coefficient sustained by the robust electronic gap, combined with the intrinsically suppressed phonon transport. Nanostructuring is typically required to open an electronic bandgap and enhance the Seebeck coefficient. Here, symmetry breaking accomplishes this electronically before any geometric confinement is invoked. The result is a system in which thermopower is amplified without sacrificing coherent transport pathways.

From a design perspective, this result marks an important conceptual shift. Conventional routes toward high thermoelectric efficiency in graphene-based materials typically rely on vacancy engineering, superlattices, isotope disorder, or hybrid edge structures to reduce thermal conductivity. In contrast, the present ribbon achieves the same objective solely through topology. The symmetry-broken lattice simultaneously reshapes the electronic spectrum, lacking electron–hole symmetry, preserves mechanical resilience, and suppresses lattice heat transport.

Thermoelectric behavior therefore emerges not as an auxiliary property layered onto a preexisting nanoribbon, but as a natural consequence of the same structural principle that governs bonding, elasticity, and electronic dispersion. The 4–5–6–8 topology transforms geometric asymmetry into a multiphysics control parameter, demonstrating that deliberate departures from hexagonal order can provide a unified pathway toward engineering coupled electronic and thermal functionalities in low-dimensional carbon systems.

Extending the multiphysical framework established above, the optical response displayed in Figure 5 reveals how the symmetry-broken bonding network governs light-matter interaction in the 4–5–6–8 nanoribbon. Optical excitations directly probe the joint density of states and the dipole-allowed interband transitions, thereby providing an integrated perspective on whether the nonequivalent polygons merely perturb the electronic band structure or reorganize it into a qualitatively distinct electronic medium. The latter clearly emerges as the physically consistent interpretation.

**Figure 5:**
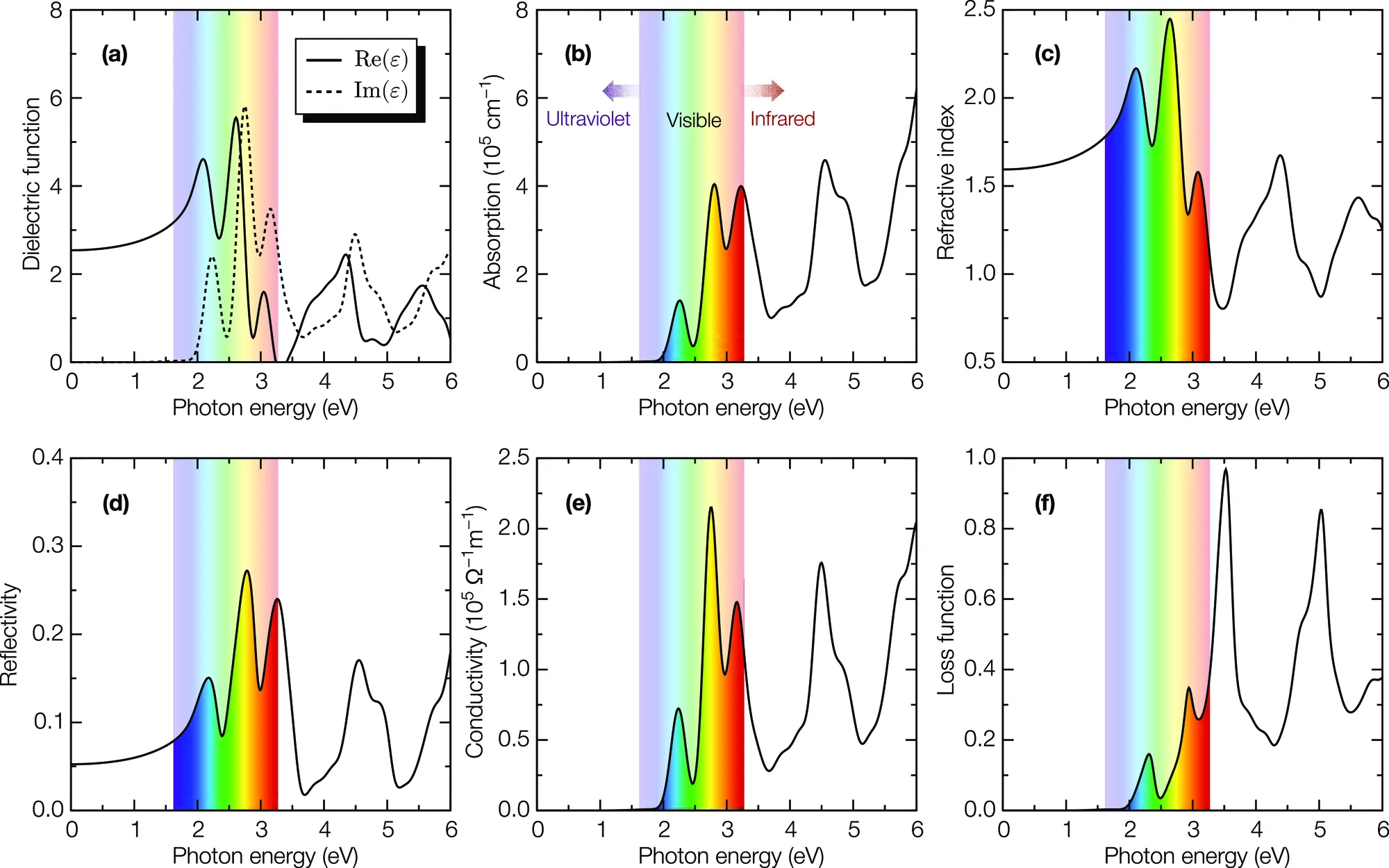
Optical properties of the 4–5–6–8 nanoribbon. (a) Real and imaginary parts of the dielectric function. (b) Absorption coefficient spanning the near-visible and ultraviolet spectral regions. (c) Refractive index. (d) Reflectivity. (e) Optical conductivity. (f) Energy loss function. The shaded region denotes the visible range, emphasizing the spectral window relevant for optoelectronic applications.

The imaginary component of the dielectric function, shown in Figure 5(a), indicates that the onset of strong optical absorption occurs near the electronic gap, defining an optical gap in the visible range. This behavior contrasts with pristine graphene, whose gapless spectrum suppresses interband absorption at low photon energies, and instead aligns the present ribbon with semiconducting graphene nanoribbons where quantum confinement activates discrete optical channels. In quasi-one-dimensional carbon systems, strongly bound excitons are known to dominate the absorption spectrum due to reduced dielectric screening and enhanced Coulomb interactions, often shifting optical transitions relative to single-particle gaps.^82^ Although the present results are obtained within an independent-particle framework, the spectral shape and sharpness of the first peaks are consistent with this confinement-driven optical landscape, reinforcing the interpretation that topology-induced band formation sets the stage for excitonic activity.

The absorption spectrum in Figure 5(b) further clarifies this point. Multiple pronounced peaks populate the visible region and extend into the ultraviolet, demonstrating that the redistribution of π-orbital overlap across nonequivalent rings generates a hierarchy of optically active transitions rather than a single threshold. Such behavior mirrors the sensitivity of graphene nanoribbon optical transitions to width, edge configuration, and family classification, parameters known to control the emergence of discrete excitonic resonances and tunable optoelectronic response.^82^ Here, however, the governing variable is not simply geometric confinement but the deliberate breaking of hexagonal symmetry. Topology, therefore, assumes a role analogous to width in conventional armchair ribbons, acting as a primary control knob for spectral engineering.

The refractive index and reflectivity, presented in Figure 5(c),(d), respectively, reinforce the presence of strong interband coupling within the visible range. Peaks in the refractive index approaching values above 2 indicate substantial electronic polarizability, whereas the moderate reflectivity suggests that incident radiation is preferentially absorbed rather than reflected. This combination is particularly desirable for nanoscale photonic and optoelectronic architectures, as it implies efficient electromagnetic coupling without excessive optical losses at interfaces.

Additional insight is obtained from the optical conductivity in Figure 5(e), whose maxima coincide with the dominant absorption features. Because optical conductivity measures the ease with which photoexcited carriers contribute to current, these peaks reveal that the same topology-enabled states responsible for band formation also support efficient photocarrier generation. In this sense, the optical response is not an isolated property but a direct continuation of the transport landscape discussed earlier, confirming that electronic delocalization extends into the excited-state regime.

Finally, the loss function in Figure 5(f) identifies collective electronic oscillations at higher photon energies, signaling the emergence of plasmon-like excitations typical of low-dimensional carbon systems. Such features originate from coherent charge-density fluctuations and are highly sensitive to the underlying band topology. Their presence suggests that the 4–5–6–8 framework may enable tunable plasmonic behavior, a possibility rarely accessible in conventional nanoribbons without external patterning or doping.

Taken as a unified optical portrait, these results demonstrate that breaking hexagonal symmetry not only opens the electronic band gap but also restructures the entire excitation spectrum, spanning single-particle transitions, photocarrier generation, and collective modes. Topology thus operates as a cross-scale design principle linking atomic bonding to macroscopic optical observables. When viewed alongside the mechanical robustness, strain-tunable electronic structure, and predictable transport response established earlier, the optical behavior confirms that the 4–5–6–8 nanoribbon constitutes a genuinely multiproperty platform in which geometry is transformed into a governing physical parameter rather than a passive structural descriptor.

## 4 Conclusions

We have demonstrated that the 4–5–6–8 carbon nanoribbon transcends the conventional interpretation of nonhexagonal rings as structural irregularities, establishing instead a materials architecture in which topology functions as a predictive design parameter. Periodic symmetry breaking reorganizes the bonding network into a stable, electronically coherent lattice that simultaneously exhibits mechanical robustness, a tunable electronic band structure, suppressed phonon transport, and strong optical activity.

Unlike standard graphene nanoribbons, where multifunctionality often relies on edge engineering, chemical modification, or extrinsic disorder, the present system embeds these capabilities directly within its geometric framework. Hybrid-functional calculations yield a band gap above 1 eV, the lattice retains a Young’s modulus of 419.4 ± 2.4 GPa, the intrinsic lattice thermal conductivity is suppressed by more than 80% relative to a width-matched 8AGNR, and the room-temperature thermoelectric figure of merit reaches ZT ≈ 0.6. Strain is converted into a controlled electronic response, thermal transport is intrinsically modulated by bond hierarchy, and optical transitions emerge from the redistributed orbital landscape. The ability of a single tight-binding parametrization to reproduce hybrid-functional trends further indicates that the governing physics is strongly determined by the underlying topology.

These findings advance a broader principle for carbon-based nanomaterials, namely that deliberately breaking hexagonal symmetry can transform geometry into a unifying physical variable that couples electronic, mechanical, thermal, and optical phenomena. The experimentally accessible 4–5–6–8 nanoribbon therefore represents more than a new member of the graphene family. It provides a blueprint for topology-driven multiproperty engineering and a pathway toward rationally designed low-dimensional systems in which functionality emerges as a direct consequence of the lattice architecture. The predictions reported here can be tested with established techniques. Bond-resolved noncontact atomic force microscopy and scanning tunneling spectroscopy can resolve the polygonal backbone and the electronic gap, angle-resolved photoemission can map the band dispersion, Raman spectroscopy can fingerprint the vibrational signatures of the tetragonal and octagonal motifs, and ultraviolet–visible absorption can probe the optical transitions.

## Data Availability

The data that support the findings of this study are available from the corresponding author upon reasonable request. The calculations were performed using the SIESTA, HONPAS, and LAMMPS packages, all openly available. Custom input files, parameter sets, and postprocessing scripts used in this study are available from the corresponding author upon reasonable request.
